# What is the Optimal Reconstruction Option after the Resection of Proximal Humeral Tumors? A Systematic Review

**DOI:** 10.2174/1874325001711010203

**Published:** 2017-03-22

**Authors:** Andrew Dubina, Brian Shiu, Mohit Gilotra, S. Ashfaq Hasan, Daniel Lerman, Vincent Y. Ng

**Affiliations:** 1 University of Maryland - Orthopaedics, Baltimore, MD, USA; 2 University of Maryland Medical Center - Orthopaedics, Baltimore, MD, USA; 3 University of Maryland Medical Center - Orthopaedics, 110 S. Paca St, 6th Floor, Baltimore, 21201, MD, USA

**Keywords:** Reconstruction, Megaprosthesis, Allograft, Shoulder, Proximal Humerus, Tumor

## Abstract

**Purpose:**

The proximal humerus is a common location for both primary and metastatic bone tumors. There are numerous reconstruction options after surgical resection. There is no consensus on the ideal method of reconstruction.

**Methods:**

A systematic review was performed with a focus on the surgical reconstructive options for lesions involving the proximal humerus.

**Results:**

A total of 50 articles and 1227 patients were included for analysis. Reoperation rates were autograft arthrodesis (11%), megaprosthesis (10%), RSA (17%), hemiarthroplasty (26%), and osteoarticular allograft (34%). Mechanical failure rates, including prosthetic loosening, fracture, and dislocation, were highest in allograft-containing constructs (APC, osteoarticular allograft, arthrodesis) followed by arthroplasty (hemiarthroplasty, RSA, megaprosthesis) and lowest for autografts (vascularized fibula, autograft arthrodesis). Infections involving RSA (9%) were higher than hemiarthroplasty (0%) and megaprosthesis (4%).

Postoperative function as measured by MSTS score were similar amongst all prosthetic options, ranging from 66% to 74%, and claviculo pro humeri (CPH) was slightly better (83%). Patients were generally limited to active abduction of approximately 45° and no greater than 90°. With resection of the rotator cuff, deltoid muscle or axillary nerve, function and stability were compromised even further. If the rotator cuff was sacrificed but the deltoid and axillary nerve preserved, active forward flexion and abduction were superior with RSA.

**Discussion:**

Various reconstruction techniques for the proximal humerus lead to relatively similar functional results. Surgical choice should be tailored to anatomic defect and functional requirements.

## INTRODUCTION

The proximal humerus is a common location for both primary and metastatic bone tumors. Numerous reconstruction and stabilization options after surgical management exist including allograft, alloprosthetic composite (APC), megaprosthesis, and more recently, reverse shoulder arthroplasty (RSA). The main goals of reconstruction are to restore function and limit complications. Patient activity, tumor characteristics, and anatomic involvement are important factors to consider when selecting the optimal reconstruction.

There is no consensus on the ideal method of reconstruction. There are numerous case series, but there is a lack of high-level comparative evidence between different options. The purpose of this study was to extensively review the existing literature.

## METHODS

**Fig. (1):**
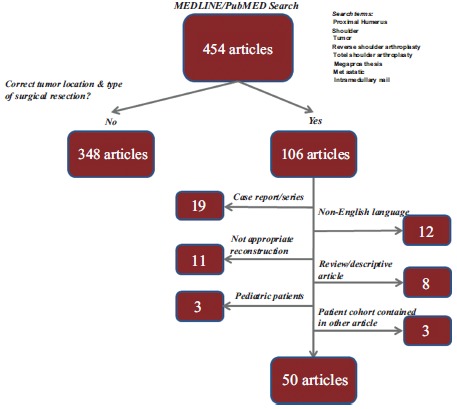
Flowchart summary of search methodology.

A systematic review of English-language literature was performed of PubMed and Medline/Ovid electronic medical databases with a focus on surgical reconstructive options for resection of proximal humerus bone lesions. All articles published as of September 1, 2015 were subject to review (Fig. **1**). Articles were excluded if they were cases series of less than 5 total patients or if scapular resection was performed. Fifty articles were included for analysis (Table **1**).

Each study was reviewed and pertinent data was recorded including patient demographics, length of follow-up, primary *versus* metastatic tumor, range of motion, rate of reoperation, infectious complications, and mechanical complications (dislocation, shoulder instability, peri-prosthetic fracture, prosthetic loosening). Post-operative functional scores were recorded when available. Data was sorted by reconstruction method. Complication rates and functional scores were calculated (Table **2**).

## RESULTS

A total of 50 articles and 1227 patients were included for analysis. The mean age of patients in the available data was 38.7 years of age with a mean post-surgical follow-up of 70.5 months. There were 30 studies qualified as Level IV evidence, 17 as Level III, and 3 studies as Level II evidence [1].

The method of reconstruction with the most published evidence was megaprosthesis. Hemiarthroplasty had relatively few articles dedicated to its use in tumor reconstruction, but likely is more widely used for humeral head lesions, particularly in metastatic scenarios. Reverse shoulder arthroplasty is a relatively newer option and has less long-term evidence than megaprosthesis or allograft reconstruction.

Mechanical complications were relatively high for all arthroplasty options ranging between 20-29%. Both allograft and autograft arthrodesis had relatively low mechanical complications (17-21%). Osteoarticular allografts had among the highest rate of mechanical complications (46%) and reoperation (34%) (Table **3**). Of the arthroplasty options, megaprosthesis had the lowest reoperation rate (10%). Infection was relatively low for both megaprosthesis (4%) and hemiarthroplasty (0%). Reverse arthroplasty had a greater than double higher infection rate (9%).

Vascularized fibula has a relatively high number of published cases. It has a low rate of infection (0%) and few mechanical complications (17%), but similar levels of reoperation (14%) to other methods of reconstruction. Claviculo Pro Humeri is a rare procedure and has very high rates of mechanical complications (47%) and infection (21%).

A variety of scores were used to assess postoperative function with the Musculoskeletal Tumor Society Score being the most consistently reported [2]. The functional outcomes were similar amongst different reconstruction options, ranging from 66% to 83%. For active range of motion, patients were generally only able obtain abduction between 45° to 90°. With resection of the rotator cuff, deltoid muscle or axillary nerve, function and stability were significantly compromised. The effect of glenoid resection varied amongst studies. If the deltoid and axillary nerve were preserved, the ability to regain active forward flexion and abduction was significantly better with RSA. To achieve external rotation with RSA, muscle transfer was occasionally necessary to compensate for a deficient posterior rotator cuff. Pain scores were not specifically reported in the majority of studies.

## DISCUSSION

Given the collection of available data from over 50 articles and 1200 patients, favorable results in most situations can be expected albeit with limited functional outcomes. Patients with extensive tumor involvement in the proximal humerus often require creative reconstruction solutions, leading to wide variability between studies and even within one institution. It should be noted that although the functional scores between reconstruction methods are similar, there is a wide spectrum of post-resection/pre-reconstruction bone and soft tissue compromise. This phenomenon can be interpreted that either a modest functional outcome is usually achievable regardless of reconstruction method or that with increasingly complex situations, increasingly complex reconstructions can achieve similar functional levels as less complex situations. If one believes the more nihilistic former approach, then it makes sense to pursue the simplest option with the least risk of complications. If one believes the latter, then reconstruction should be tailored to the specific anatomic scenario with some consideration to the patient’s physical demands and tolerance for complications. Because numerous reconstructive options are available, adequate margins should always be endeavored based on the clinical situation in order to minimize the risk of local recurrence, particularly for more aggressive phenotypes. For patients with limited estimated lifespan such as in the setting of metastatic disease and in situations which postoperative radiation and chemotherapy are required, reconstructive options that allow early weight bearing and use of the shoulder and that do not rely on bone healing such as prosthetic replacement are preferred.

Hemiarthroplasty is useful for minor bone loss situations such as primary malignant tumors limited to the humeral head and metastatic lesions not amenable to intramedullary nailing. Because shoulder function with hemiarthroplasty is dependent on the integrity of the rotator cuff and greater tuberosity which is often compromised by tumor involvement it is not surprising that functional scores are limited. Although the mechanical complication rate was relatively high in this systematic review, it can be partially attributed to the frequency of subluxation requiring soft tissue reconstruction [3, 4]. Glenoid wear can be expected in young patients with hemiarthroplasty [5], but most oncological patients requiring hemiarthroplasty are >50 years old. Conversion of a painful hemiarthroplasty to total shoulder arthroplasty lead to a high rate of unsatisfactory results [6].

Current revision long-stem humeral stems allow surgeons to cement a hemiarthroplasty slightly proud and compensate for a limited bone defect of the medial calcar. For more extensive bone loss, megaprostheses are a relatively simple solution. This study group of over 700 patients includes a variety of prostheses and pre-reconstruction bone deficits. Consequently, there is a wide spectrum of functional MSTS scores in this group (55-82%). Active range of motion after megaprosthesis reconstruction is largely dependent on healing of the tendon-prosthetic interface, which is unpredictable at best. Nevertheless, the overall complication rates for megaprostheses were relatively favorable with limited infections (4%), revision surgery (10%) and mechanical complications (17%). No study had an infection rate greater than 10% and multiple studies reported 0% [7-10]. Additionally, mechanical complications were commonly treated conservatively including subluxation, dislocation, prosthetic loosening, and periprosthetic fracture.

The clinical context for RSA is unclear. Situations in which sacrifice of the rotator cuff is necessary but preservation of the deltoid insertion and axillary nerve is possible, RSA may be considered. For non-oncological situations, RSA is conventionally reserved for older, lower-demand patients because longevity of modern implants are unknown and there is risk of a ‘tired deltoid’ at ten years [11]. It is also often reported to have a higher complication rate than other arthroplasty options [12]. For oncologic patients, an older, lower-demand demographic is typically an indication for less functionally aggressive options such as megaprosthesis or hemiarthroplasty. Many elderly oncology patients require their upper extremities to push oneself out of a chair or to support themselves due to lower extremity weakness. This motion (extension, adduction, external rotation, axial loading) predisposes them to dislocation of RSA. Additionally, the higher infection rate (9%) may delay postoperative chemotherapy or radiation therapy. For younger individuals, they will likely encounter many or more of the same complications with RSA as young non-oncologic patients.

For high demand, younger patients requiring resection distal to the deltoid insertion, an alloprosthetic composite (APC) may be advantageous to allow for tendinous reattachment to preserved allograft tendon insertions. However, APC is a technically more challenging procedure and has a much higher rate of complications requiring revision than megaprosthesis including fracture and nonunion. The functional results for APC are similar to other reconstructive options and so the risks and benefits need to be carefully weighed.

Osteoarticular allografts are less frequently used since the advent of improved prosthetic options. With a high rate of complications requiring reoperation, numerous fractures and a lengthy time to union, there are no highly compelling reasons to choose osteoarticular bulk allografts in oncologic situations. Several articles report rates of mechanical failure in over 60% of cases [13-16]. Autologous vascularized fibular grafts, with or without allograft supplementation, on the other hand, have superior results and fewer complications. If early fracture is avoided, the graft has the ability to hypertrophy, to avoid infection and unite with the native bone to a greater extent than allograft or non-vascularized autograft. Claviculo-Pro-Humeri (CPH) similarly provides a biologic reconstruction option as the ipsilateral clavicle functions as a rotational bone flap to replace the resected proximal humerus. Its principle advantage is the construct’s inherent proximal stability through the acromioclavicular ligaments. It reportedly has the best functional outcomes of all reconstruction options, but is limited to pediatric patients and may often require reoperation for nonunion [17].

Arthrodesis is traditionally limited to young adult patients expected to subject their shoulders to high levels of physical stress and to patients undergoing salvage of a failed limb-sparing reconstruction. Both allograft and autograft options appear to yield similar rates of mechanical complications and infection. Remarkably, functional scores are also similar to other motion-preserving reconstructions and are similar between primary or secondary arthrodesis [18]. Patients are able to compensate through preserved scapulothoracic and elbow motion.

In conclusion, hemiarthroplasty is the simplest option for minimal bone loss. For loss of the rotator cuff and deltoid insertion/axillary nerve, RSA and APC, respectively, provide potential for greater function, but have higher complications than megaprosthesis and the risks and benefits need to be carefully considered. Autograft arthrodesis, vascularized fibula, and CPH are effective in certain situations.

## Figures and Tables

**Table 1:** Summary of data from literature review on proximal humeral reconstruction.

Authors	Type of Fixation	"n"	Mean Follow-up (mo)	Mean age (yrs)	Tumor Type (% primary lesions)	Tumor-specific Mortality
Salzer M *et al.* (1979) [19]	Megaprosthesis	27	27.4		58%	37%
Campanacci M *et al.* (1982) [20]	Megaprosthesis	13			85%	31%
Bos G *et al.* (1987) [10]	Megaprosthesis	18	68.4		100%	11%
Ross AC *et al.* (1987) [21]	Megaprosthesis	19	132		89%	11%
Capanna R *et al.* (1988) [22]	Megaprosthesis	19	18.2	51	0%	63%
Gebhardt MC *et al.* (1990) [23]	Osteoarticular allograft	23	63.6	33	96%	13%
Jensen RL *et al.* (1995) [24]	*Overall*	19	39		100%	23%
	APC	4		43	100%	0%
	Hemiarthroplasty	15		38	100%	27%
O'Connor MI *et al.* (1996) [13]	*Overall*	20			100%	
	Osteoarticular allograft	8				
	Megaprosthesis	11				
	Allograft arthrodesis	1				
Freedman *et al.* (1997) [25]	Megaprosthesis	5			20%	60%
Probyn LJ *et al.* (1998) [26]	*Overall*	21				
	Osteoarticular allograft	11	45.6	34	100%	0%
	Allograft arthrodesis	7				
	Autograft arthrodesis	3				
Asavamongkolkul A *et al.* (1999) [27]	Megaprosthesis	59	90	33	90%	46%
Fabroni RH *et al.* (1999) [8]	Megaprosthesis	8	165	22	100%	
Getty PJ *et al.* (1999) [14]	Osteoarticular allograft	16	34			13%
Wada T *et al.* (1999) [28]	Vascularized fibula	8	70	27	100%	13%
Shin KH *et al.* (2000) [29]	*Overall*	7	35.6	23.4		18%
	Megaprosthesis	1				
	APC	6				
Gebhart M *et al.* (2001)[7]	Megaprosthesis	16				
Rodl W *et al.* (2002)[30]	*Overall*	45		27	100%	36%
	Osteoarticular allograft	11		20	100%	
	CPH	15		18	100%	
	Megaprosthesis	19		37	100%	
De Wilde L *et al.* (2003) [31]	RSA	13	36	48.8		
Ippolito V *et al.* (2003) [32]	Megaprosthesis	20		68	0%	
Kumar D *et al.* (2003) [33]	Megaprosthesis	100	108	34	83%	44%
DeGroot H *et al.* (2004) [16]	Osteoarticular allograft	32		30		6%
Zeegen EN *et al.* (2004) [34]	Megaprosthesis	15		49		
Fuchs B *et al.* (2005) [18]	*Overall*	21	231	26		0%
	Allgraft arthrodesis	12	123.6	26		0%
	Autograft arthrodesis	9	157	27		0%
Mayilvahanan N *et al.* (2006) [35]	Megaprosthesis	57	66	27.9	91%	11%
Black AW *et al.* (2007) [36]	APC	6	55		83%	
Kitagawa Y *et al.* (2007) [4]	*Overall*	6	21	54	100%	32%
	Hemiarthroplasty	5	38	55	100%	
	Allograft arthrodesis	1		51	100%	
Sharma S *et al.* (2007) [9]	Megaprosthesis	21	47.9			
El-Sherbiny M *et al.* (2008) [37]	*Overall*	32		21	97%	6%
	Megaprosthesis,	13				
	Vascularized fibula	11				
	Pedicled lateral scapular crest graft	8				
Scotti C *et al.* (2008) [38]	Megaprosthesis	40		67	0%	
Cannon CP *et al.* (2009) [39]	Megaprosthesis	83	30	55		
Moran M *et al.* (2009) [3]	Hemiarthroplasty	11	69	21.5	100%	18%
Potter B *et al.* (2009) [15]	*Overall*	49	113	48.5	51%	51%
	Osteoarticular allograft	17		36.5		
	APC	16		56.3		
	Megaprosthesis	16		53.6		
Piccioli A *et al.* (2010) [40]	Megaprosthesis	30			0%	
Raiss P *et al.* (2010) [41]	Megaprosthesis	39	38		23%	23%
Wang Z *et al.* (2010) [42]	*Overall*	25	48	32	88%	8%
	Osteoarticular allograft	12				
	APC	7				
	Megaprosthesis	6				
Yang Q *et al.* (2010) [43]	*Overall*	12			100%	
	Osteoarticular allograft	3				
	Megaprosthesis	7				
	Vascularized fibula	2				
De Wilde L *et al.* (2011) [44]	RSA	14	92.4	45.1	71%	29%
Griffiths D *et al.* (2011) [45]	Megaprosthesis	58	71	46	59%	28%
Ruggieri P *et al.* (2011) [46]	APC	14	25	35	100%	0%
Bilgin SS (2012) [47]	Autograft arthrodesis	6	60			
Hartigan DE *et al.* (2012) [48]	APC	27	76.8	43.8	85%	11%
Li J *et al.* (2012) [49]	Vascularized fibula	6	19.1	15.8	100%	0%
Aponte-Tinao LA *et al.* (2013) [50]	*Overall*	37	60	32		
	Osteoarticular allograft					
	APC					
Kaa AK *et al.* (2013) [51]	RSA	16	46	41.5	50%	31%
van de Sande *et al.* (2013) [52]	*Overall*	37	120	44.8	89%	27%
	Osteoarticular allograft	13				46%
	APC	10				20%
	Megaprosthesis	14				14%
Liu T *et al.* (2014) [53]	*Overall*	41	57.7	30.6	100%	
	Megaprosthesis	25				32%
	Vascularized fibula	16				38%
Bonnevialle N *et al.* (2015) [54]	RSA	10	42	55	60%	20%
Calvert GT *et al.* (2015) [17]	CPH	4		5.9		
Pruksakorn D *et al.* (2015) [55]	Megaprosthesis	13	14.3		0%	15%
Streitbuerger A *et al.* (2015) [56]	Megaprosthesis	18	33.6	42	66%	11%

RSA: Reverse Shoulder Arthroplasty, APC: Alloprosthetic Composite, CPH: Claviculo Pro Humeri

**Table 2:** Summary of reconstruction techniques and complications.

Treatment Method	Number of Articles	'n'	Average Age (yrs)	Infection Rate (%)	Mechanical Failure (%)	Reoperation (%)	MSTS (%)
Reverse shoulder arthroplasty	4	53	47	9%	23%	17%	74%
Hemiarthroplasty	4	31	35	0%	29%	26%	66%
Megaprosthesis	30	761	45	4%	17%	10%	72%
Alloprosthetic composite	9	106	45	6%	30%	26%	73%
Osteoarticular allograft	11	167	31	7%	46%	34%	74%
Vascularized fibula	5	43	22	0%	17%	14%	73%
Allograft arthrodesis	4	19	26	12%	21%	32%	74%
Autograft arthrodesis	3	20	25	7%	17%	11%	76%
CPH	2	19	18	21%	47%	47%	83%
Pedicled lateral scapula graft	1	8			25%	25%	68%
Total	50	1227					

**Table 3:** Summary of mechanical complications.

	Mechanical Complication (% of total)	Re-operation (% of total)
Instability (subluxation, dislocation)	52.0	24.1
Aseptic loosening	10.5	16.1
Non-union	23.0	12.6
Fracture	9.9	26.4
Infection		17.8
Other	4.6	2.9

## LIST OF ABBREVIATIONS

APC: = Alloprosthetic Composite

RSA: = Reverse Shoulder Arthroplasty
